# Wheat Seed Detection and Counting Method Based on Improved YOLOv8 Model

**DOI:** 10.3390/s24051654

**Published:** 2024-03-03

**Authors:** Na Ma, Yaxin Su, Lexin Yang, Zhongtao Li, Hongwen Yan

**Affiliations:** College of Information Science and Engineering, Shanxi Agricultural University, Taigu District, Jinzhong 030801, China; 19935907396@163.com (Y.S.); 18734840526@163.com (L.Y.); 18835497995@163.com (Z.L.); yhwhxh@126.com (H.Y.)

**Keywords:** wheat seed detection, YOLOv8, lightweight, attention mechanism

## Abstract

Wheat seed detection has important applications in calculating thousand-grain weight and crop breeding. In order to solve the problems of seed accumulation, adhesion, and occlusion that can lead to low counting accuracy, while ensuring fast detection speed with high accuracy, a wheat seed counting method is proposed to provide technical support for the development of the embedded platform of the seed counter. This study proposes a lightweight real-time wheat seed detection model, YOLOv8-HD, based on YOLOv8. Firstly, we introduce the concept of shared convolutional layers to improve the YOLOv8 detection head, reducing the number of parameters and achieving a lightweight design to improve runtime speed. Secondly, we incorporate the Vision Transformer with a Deformable Attention mechanism into the C2f module of the backbone network to enhance the network’s feature extraction capability and improve detection accuracy. The results show that in the stacked scenes with impurities (severe seed adhesion), the YOLOv8-HD model achieves an average detection accuracy (mAP) of 77.6%, which is 9.1% higher than YOLOv8. In all scenes, the YOLOv8-HD model achieves an average detection accuracy (mAP) of 99.3%, which is 16.8% higher than YOLOv8. The memory size of the YOLOv8-HD model is 6.35 MB, approximately 4/5 of YOLOv8. The GFLOPs of YOLOv8-HD decrease by 16%. The inference time of YOLOv8-HD is 2.86 ms (on GPU), which is lower than YOLOv8. Finally, we conducted numerous experiments and the results showed that YOLOv8-HD outperforms other mainstream networks in terms of mAP, speed, and model size. Therefore, our YOLOv8-HD can efficiently detect wheat seeds in various scenarios, providing technical support for the development of seed counting instruments.

## 1. Introduction

Wheat is one of the main crops in the world today, with about 40% of the population relying on wheat as their main food source [1]. China is the largest producer and consumer of wheat globally, and improving wheat yield and quality is of great significance for food security [2,3]. Thousand-grain weight is an important indicator for evaluating wheat quality and yield [4,5,6,7]. Thousand-grain weight is measured in grams and represents the weight of 1000 grains [8,9]. However, accurate counting of wheat grains is necessary to determine thousand-grain weight. Therefore, precise detection of wheat seeds is crucial. Seed detection can also provide important support for wheat breeding, phenotype analysis, sorting damaged and moldy grains, and other purposes. However, in complex agricultural production environments, there are challenges such as overlapping and dense adhesion of wheat seeds, which greatly affect counting accuracy. Achieving accurate detection of wheat seeds under adhesion has become a hot topic in related research and is receiving increasing attention.

In the early days, wheat seeds were mostly counted manually, which could not meet the demand. The counting work was tedious and required patience. It could only be accurately counted under extreme concentration. Prolonged work could easily tire the human eyes, posing a great challenge. Additionally, it was difficult to identify the standards for high-quality wheat seeds, resulting in high labor costs and large errors in this counting method. Although seed counters were introduced to solve the problems of traditional manual counting in field experiments, with higher accuracy and automation effectively liberating a large amount of manpower, the issue of slow identification speed of seed counters in large-scale field yield estimation experiments has also emerged. Therefore, maintaining a high accuracy rate while simultaneously having faster detection speed for counting and statistics has always been a topic of discussion among scholars.

In recent years, machine vision technology has been continuously developed and improved, and it has been widely used in research on crop quality inspection, yield estimation, and seed counting. Based on this, researchers have proposed seed counting methods based on traditional digital image processing [10,11,12,13].

Zhao et al. [14] proposed a new method for improving the efficiency and accuracy of seed counting using MATLAB image processing techniques and mechanical vibration technology. It effectively addresses the issues of seed overlap and impurity through mechanical vibration and image erosion processing. Zhao et al. [15] developed an automated method for counting corn kernels based on image processing. They proposed an image preprocessing method according to the characteristics of corn cob images. This method includes median filtering to eliminate random noise, Wallis filtering to sharpen image edges, and histogram enhancement. The accuracy of kernel counting for corn cobs using this method can reach 96.8%. Wu et al. [16] compared several methods using different image acquisition devices and various shapes of panicles for counting accuracy in indica and japonica subspecies of rice. The developed linear regression model achieved counting accuracies of over 96% and 97% for japonica and indica rice seeds, respectively.

However, along with the advantages of traditional image processing techniques in seed counting, there are also some disadvantages. Traditional image processing techniques require manual extraction of seed features, which is difficult in practical applications. It is heavily influenced by human factors and requires a significant amount of time and effort. Additionally, the limitations of traditional image processing models restrict their operation to specific environments and experimental conditions. For example, if there is a change in the height of the captured seed image, the model may fail to recognize the seeds, making it challenging to apply in real-world seed detection and yield estimation applications.

With the development of high-performance computer hardware, deep learning has become a research hotspot. Deep learning algorithms for various processing tasks are constantly improving and innovating and are being applied in various fields [17,18,19,20]. The integration and innovation of traditional agricultural production methods with deep learning has become a general trend, and agricultural informatization and intelligentization have been vigorously developed. Currently, deep learning is widely applied in fields such as plant disease and insect pest control [21,22,23], plant counting [24,25,26,27], and plant phenotyping [28,29,30,31].

Deng et al. [32] proposed the seed detection model for automatically identifying and counting seeds on the main branch of rice panicles. This model uses an image analysis approach based on deep learning convolutional neural networks (CNNs) and integrates feature pyramid networks (FPNs) into the faster R-CNN network. The overall accuracy of the grain detection model was 99.4%. Li et al. [33] utilized annotated information to generate ground truth density maps using convolutional Gaussian kernels. They designed a simple and effective method, using a dual-column convolutional neural network (TCNN) to interpret pod images into seed density maps, ultimately achieving seed counting. The mean absolute error (MAE) was 13.21, and the mean squared error (MSE) was 17.62. Devasena et al. [34] proposed a new quality checking process through a machine vision system with deep learning. The seeds are passed through cameras, and image process techniques with deep learning algorithms are utilized to match the quality, which is trained into the system to identify and classify the seeds. Shi et al. [35] utilized an improved lightweight object detection method, YOLOv5s-T, to detect and count wheat spikes. The coefficient of determination (R^2^) between the predicted and true values of wheat spikelets was 0.97 for the flowering stage, 0.85 for the grain filling stage, and 0.78 for the mature stage. Feng et al. [36] used two deep learning-based counting algorithms for rice: an MCNN-based algorithm and a density map-based counting algorithms. Additionally, they introduced an improved algorithm with advanced priors based on the original algorithm. After the experiments, it was proven that both algorithms can count rice well. Sun et al. [37] proposed a deep learning optimization method based on pre-labeling contour grouping for counting overlapping rice seeds. The average error rate for rice seeds in a single image was 1.06%, and the average recognition time of counting was 0.45 s.

The above studies mainly focus on the occlusion problem between the target and complex background. When multiple targets are occluded and the degree of occlusion is high, only very small local features are visible. The algorithms used in these studies cannot accurately identify the target from the remaining unoccluded local features alone. As a result, occluded targets may be mistakenly recognized as the same targets as other adjoining targets, leading to missed detections. In addition, most of the seed detection methods based on deep learning currently have high detection accuracy but also high computational complexity, resulting in slow detection speed. On the other hand, methods with low computational complexity and fast detection speeds often sacrifice detection accuracy. This is because the computational resources on the embedded platform of the seed counter are limited. The slow detection speed of complex models cannot meet real-time requirements, which poses challenges in deployment. Therefore, finding a balance between detection speed, detection accuracy, and model computational complexity in seed detection methods has always been a hot and challenging research topic.

We evaluated the recent popular deep learning network, YOLOv8, as the latest detection algorithm in the YOLO family. It has the characteristics of high detection efficiency, high accuracy, and small model memory occupation. Therefore, based on YOLOv8 as a benchmark, we proposed a lightweight real-time wheat seed detection model called YOLOv8-HD, focusing on detecting wheat seeds in different scenarios. Our contributions are summarized as follows:

- We created a well-labeled dataset of wheat seeds. The dataset includes five different scenarios: dispersed without impurities, dispersed with impurities, aggregated without impurities, aggregated with impurities, and stacked, covering the placement of wheat seeds in various situations, which helps in counting the number of wheat seeds in different scenarios.
- Based on YOLOv8, we designed a lightweight detection method using the idea of shared parameters. To improve detection accuracy, we incorporated the Vision Transformer with Deformable Attention mechanism into the C2f module. Finally, we proposed a lightweight real-time YOLOv8-HD model for wheat seed detection and performed statistical counting of the detected wheat seeds.
- We conducted extensive experiments on wheat seed detection tasks, and the results showed that our proposed YOLOv8-HD model, compared to other detection algorithms, not only improved detection accuracy but also further reduced model size and improved inference speed, providing technical support for real-time counting of wheat seeds on embedded platforms.

## 2. Materials and Methods

### 2.1. Dataset Processing

#### 2.1.1. Dataset

The wheat seed used in this study is Changmai 6197. This seed has a compact plant type, good stem elasticity, and is resistant to drought, lodging, freezing, premature senescence, and has high and stable yields. It is a new variety of drought-resistant and high-yielding wheat, suitable for dryland cultivation in the central part of Shanxi Province, China. The wheat seed image data in this study were taken using a Vivo Z3i smartphone, and the wheat seeds were randomly placed in each batch.

Traditional wheat seed segmentation algorithms mainly focus on segmenting 2–20 adhered seeds, and the segmentation effect is not satisfactory for more adhered seeds. Therefore, based on previous research results [38,39,40,41], this study defines the local region containing 2–20 adhered seeds as mild adhesion and the local region containing more than 20 adhered seeds as severe adhesion. To enable the model to learn more features of adhered wheat seeds, as many wheat seed images as possible should be input for training, and the images should include both mild and severe adhesion of wheat seeds. Therefore, in the experiment, a certain number of wheat seeds were randomly scattered on the platform, and slight shaking was performed to make the wheat seeds distribute randomly, preventing the occurrence of single adhesion situation images due to human intention.

**Figure 1:**
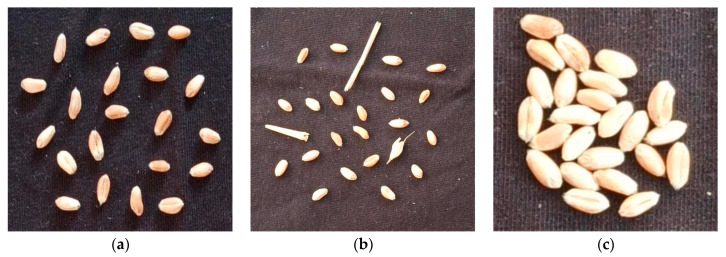

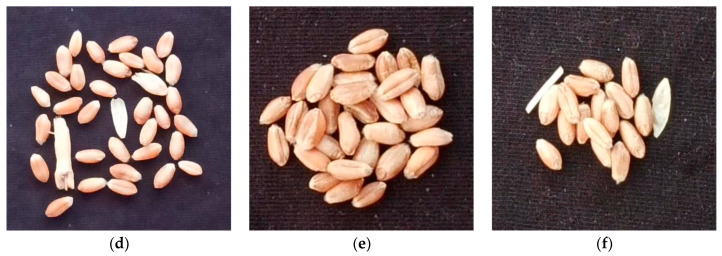
Wheat seed collection example images: (**a**) scattered without impurities; (**b**) scattered with impurities; (**c**) clustered without impurities; (**d**) clustered with impurities; (**e**) stacked without impurities; (**f**) stacked with impurities.

Finally, this study set up five scenarios: dispersed without impurities, dispersed with impurities, aggregated without impurities, aggregated with impurities, and stacked. Data were collected with 100 images for each scenario, totaling 500 images. The specific divisions of the five scenarios are presented in Table 1:

Example images collected under different scenarios are shown in Figure 1.

#### 2.1.2. Dataset Labeling

Using LabelImg software, different wheat seeds, husks, and straws in the images were labeled. The labeling format is in .txt documents, with the wheat seed labeled as “w”, the husk labeled as “k”, and the straw labeled as “g”. Due to the lower presence of husks and straws as impurities in the wheat seeds, there are fewer labeled instances of husks and straws in the collected images. Therefore, there is a severe data imbalance, with a higher number of labeled instances for wheat seeds. This situation requires higher demands for wheat seed detection algorithms.

The numbers of different categories labeled in the dataset are shown in Table 2.

#### 2.1.3. Dataset Augmentation

**Figure 2:**
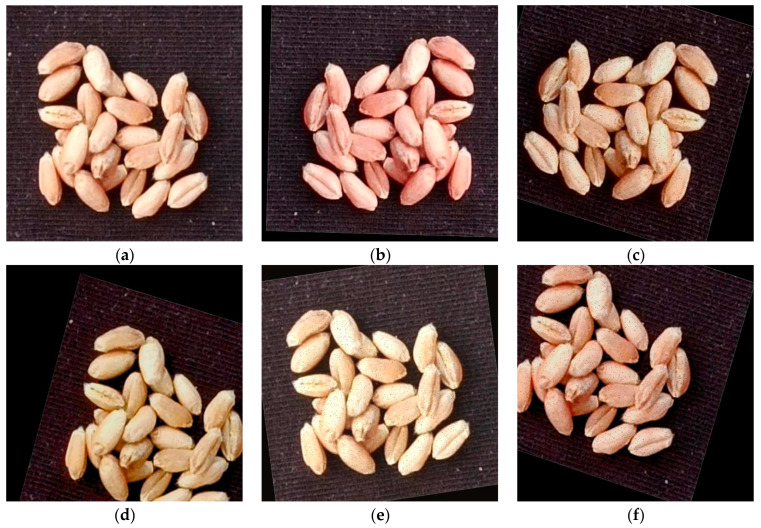
Wheat seed data augmented images: (**a**) original image; (**b**) augmented image 1; (**c**) augmented image 2; (**d**) augmented image 3; (**e**) augmented image 4; (**f**) augmented image 5.

In order to improve training model performance and enhance model generalization, data augmentation techniques are used to increase the number of samples and prevent overfitting caused by insufficient training data. In this study, random pixel removal, image sharpening, affine transformation, brightness adjustment, hue adjustment, and horizontal flipping are randomly combined as data augmentation methods to expand the dataset. Five new augmented images are generated for each original image. Some examples of data augmentation samples are shown in Figure 2.

A total of 3000 sample images were obtained through data augmentation, and they were randomly divided into a training set, a validation set, and a test set in a ratio of 7:2:1. The specific division of the training set, validation set, and test set for the five scenes is shown in Table 3.

After dataset partitioning, the annotation counts of different categories in the five scenes are shown in Table 4.

### 2.2. Improved YOLOv8-HD Network

Wheat seed counting is prone to interference from impurities such as wheat straw and husks, especially when some husks are similar to wheat seeds and are easily mistaken for seeds. Additionally, wheat seeds are susceptible to adhesion and stacking, making accurate detection of wheat seeds challenging. Existing deep learning-based convolutional neural network models achieve high detection accuracy but come with high computational complexity and slow detection speed. To balance detection speed, accuracy, and computational complexity, as well as effectively address the issue of impurities and seed stacking affecting detection performance, this study improves the YOLOv8 model.

**Figure 3:**
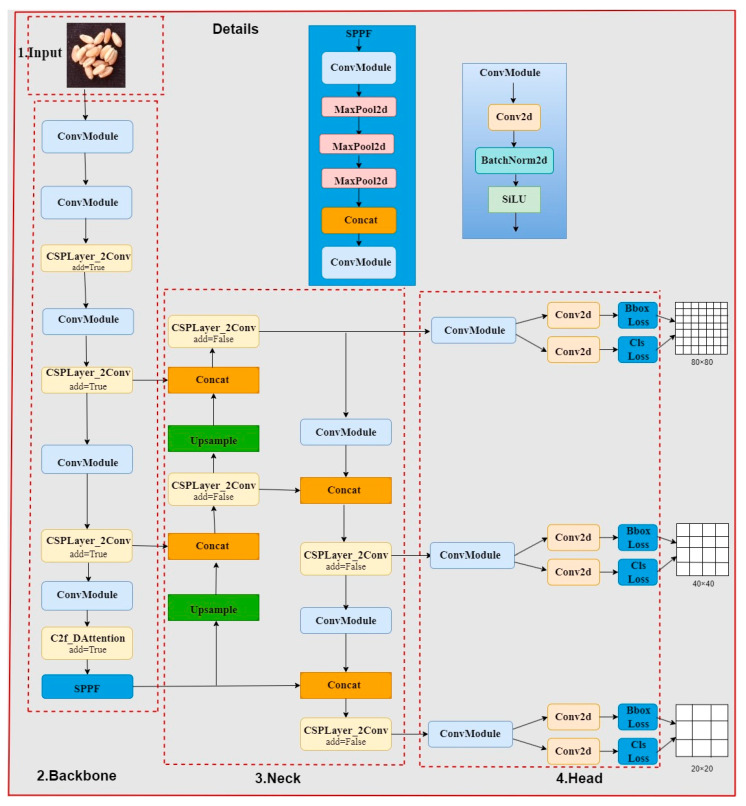
Improved YOLOv8 lightweight network model.

Firstly, to achieve high detection accuracy and speed with minimal model parameters, the detection head of YOLOv8 is designed to be lightweight, sharing the convolutional layer. To enhance wheat seed detection performance under the presence of impurities and seed stacking, a Vision Transformer with Deformable Attention mechanism is integrated into the C2f module of the backbone network to improve network feature extraction capabilities. We named the improved YOLOv8 model YOLOv8-HD. The lightweight wheat seed detection model structure of YOLOv8-HD is shown in Figure 3.

#### 2.2.1. Lightweight Design of Detection Head

**Figure 4:**
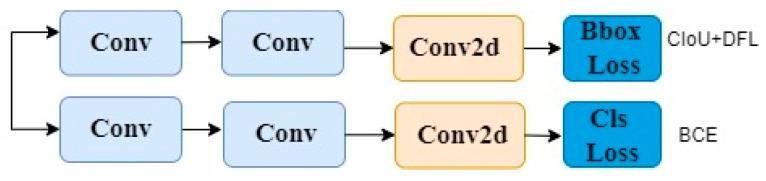
YOLOv8 head.

The head of YOLOv8 adopts the currently mainstream Decoupled-Head structure, separating the classification and detection heads. The head of YOLOv8 first branches into two 3 × 3 convolutional modules, then each goes through a Conv2d module, and finally calculates the Cls loss and Bbox loss separately. The design of the YOLOv8 detection head is shown in Figure 4.

**Figure 5:**
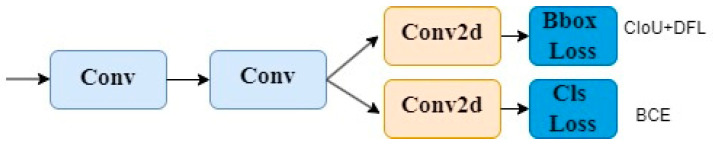
Lightweight YOLOv8 detection head.

Due to the small size of wheat seeds as the target, in order to improve detection speed and reduce the parameter quantity of YOLOv8, we made lightweight design modifications to the detection head of YOLOv8. It is modified to first share a 1 × 1 convolutional layer and a 3 × 3 convolutional layer. Then, each goes through a Conv2d module, and finally calculates the Cls loss and Bbox loss separately. The modified detection head structure is shown in Figure 5.

#### 2.2.2. Vision Transformer with Deformable Attention

The Vision Transformer with Deformable Attention (DAT) is a simple yet effective deformable self-attention module proposed by Zhuofan Xia et al. in 2022 [42]. This module selects the positions of key-value pairs in self-attention in a data-dependent manner. This flexible approach allows the self-attention module to focus on relevant regions and capture more information. A powerful Pyramid Backbone, called the Deformable Attention Transformer (DAT), is constructed on this module for image classification and various dense prediction tasks. Therefore, in this study, DAT is integrated into the YOLOv8 backbone network C2f to better extract wheat seed features.

Compared to CNN models, Transformer-based models have a larger receptive field and are adept at modeling long-term dependencies. They have achieved excellent performance with a large amount of training data and model parameters. However, they come with higher computational costs, slower convergence speed, and increased risk of overfitting. In order to reduce computational complexity, Swin Transformer adopts window-based local attention to restrict attention within a local window, while Pyramid Vision Transformer (PVT) saves computational resources by down-sampling key and value feature maps. However, manually designed attention mechanisms are data-agnostic. For a given query, we expect its key/value set to be flexible and adjustable according to different inputs. The success of Deformable Convolution Networks (DCNs) has prompted the exploration of deformable attention patterns in Vision Transformers. However, due to high computational costs, no one has considered it as a basic component for building a powerful backbone. DAT is a simple and efficient deformable self-attention module that can capture more informative features.

**Figure 6:**
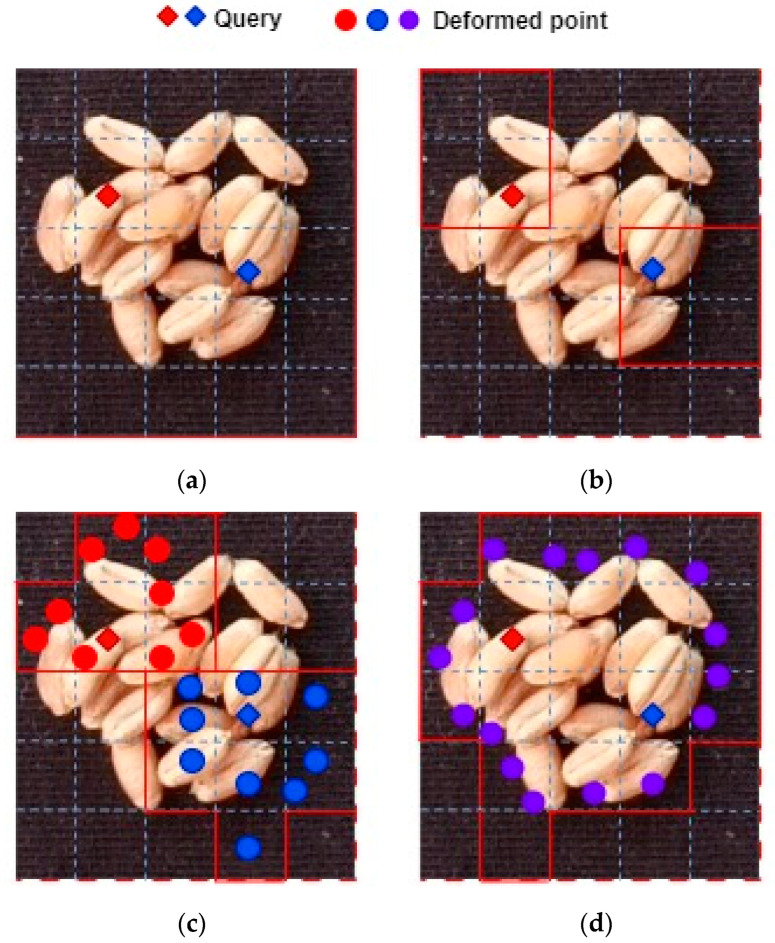
Comparison between DAT and other Vision Transformer models: (**a**) VIT; (**b**) Swin Transformer; (**c**) DCN; (**d**) DAT.

The comparison between DAT and other visual transformer models is shown in Figure 6:

- In ViT, all Q have the same receptive field, targeting global features for all positions.
- In Swin, there is local attention, so the receptive field regions for two Q in different windows are different.
- DCN learns biases for the surrounding nine positions and then samples and corrects the feature positions. As shown in the figure, there are a total of nine red and blue points.
- DAT combines ViT and DCN. All Q share the same receptive field, but these receptive fields have learned positional biases. To reduce computational complexity, the number of targeted features is also down-sampled. Therefore, there are a total of 16 sampling points in the figure, which is 1/4 smaller than the original.

**Figure 7:**
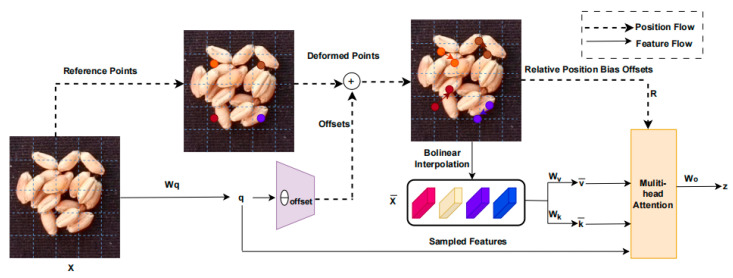
Information flow of the deformable attention mechanism in DAT.

The information flow of the deformable attention mechanism in DAT is shown in Figure 7. A set of reference points is uniformly placed on the feature map, and its offsets are learned from the queries through an offset network. Then, the deformed keys and values are projected from the sampled features based on the deformation points.

**Figure 8:**
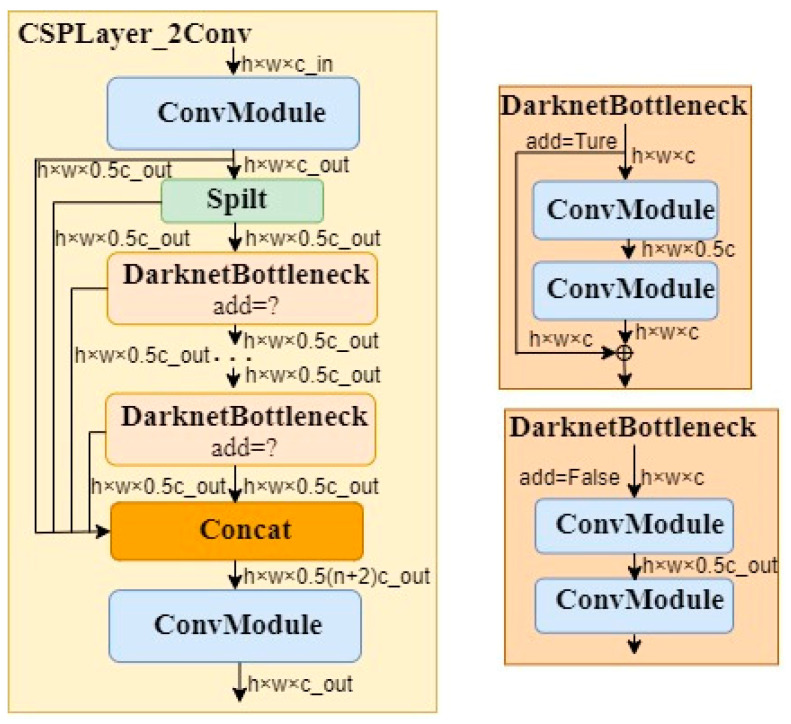
C2f model.

The C2f structure is shown in Figure 8. In Figure 8, we can see that the C2f module first goes through Conv, enters the Split module, goes through multiple DarknetBottleneck modules, then enters the Concat module, and finally goes through the Conv module for output. The DarknetBottleneck module has two forms, as detailed in Figure 8.

To enhance the feature extraction capability of the C2f module, we integrated the DAT attention mechanism after the two convolutional layers in the DarknetBottleneck module. Then, we replaced the C2f module in the 8th layer of the original YOLOv8 with the C2f module incorporating the DAT mechanism. The YOLOv8 backbone network parameters after the improvement are shown in the Table 5.

**Figure 9:**
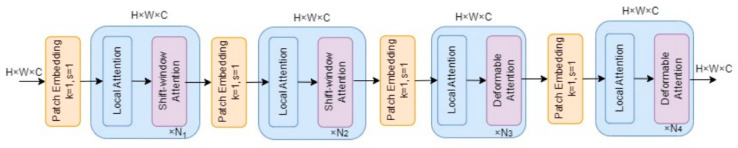
DAT network parameters.

From Table 5, it can be seen that the channel parameter of the 8th layer is 256, with an image size of 20 × 20. Upon entering the C2f module, it goes through a Split channel to become 128 channels. When entering the Bottleneck module, it undergoes two Conv operations, then enters the DAT network, where both the output channels and image size remain unchanged. It then goes through the Concat module, and finally through a Conv module, with an output parameter of 20 × 20 × 256. The parameters of the DAT network are set, as shown in Figure 9.

### 2.3. Evaluation Metrics

The process of wheat seed detection requires consideration of both detection accuracy and speed. Therefore, this study adopts precision, recall, Average Precision (AP), and mean Average Precision (mAP) metrics to characterize the performance of the models. Additionally, the models’ running speed is evaluated using metrics such as GFLOPs (Giga Floating-point Operations Per second).

Precision is the proportion of cases that are classified as positive and are actually positive in the entire sample. Accuracy is calculated using Formula (1):

(1) $$Precision=\frac{TP}{TP+FP}$$

Recall represents the proportion of actual positive cases to the predicted positive cases. Recall is calculated using Formula (2):

(2) $$Recall=\frac{TP}{TP+FN}$$

where:

TP represents the number of correct predictions as positive samples.

FP represents the number of incorrect predictions as positive samples.

FN represents the number of incorrect predictions as negative samples.

AP (Average Precision) represents the area under the precision–recall curve enclosed by the curve and the coordinate axis. It is calculated using Formula (3):

(3) $$AP=\int_{0}^{1}\left(Precision\times Recall\right)dx$$

mAP (mean Average Precision) represents the average AP value for three categories in this study, namely wheat seeds, wheat stems, and wheat husks. It is calculated using Formula (4):

(4) $$mAP=\sum_{i=1}^{i=3}{AP}_{i}/3$$

The mAP0.5:0.95 is the average mAP (mean Average Precision) calculated based on ten different IoU (Intersection over Union) thresholds. These thresholds range from 0.5 to 0.95, with a step size of 0.05.

To further evaluate the algorithm’s performance and analyze the network’s feature extraction capabilities in more detail, TIDE, a framework and related toolbox for analyzing error sources in object detection and instance segmentation algorithms, is used.

TIDE defines six error types:

Classification error (Cls): Correct localization but incorrect classification.

Localization error (Loc): Correct classification but incorrect localization.

Both classification and localization errors (Both): Both classification and localization are incorrect.

Duplicate detection error (Dupe): Correct classification, but another detection with a higher score has matched the target. In other words, it is correct if there is no detection with a higher score.

Background error (Bkg): Background detected as foreground.

Miss undetected error (Miss): All ground truths that were not detected except for Cls and Loc errors.

### 2.4. Experiment and Model Training

The operating system used for the experiment is Windows 10. The CPU model is Intel(R) Core(TM) i7-13700F @2.10GHz. The GPU model is NVIDIA GeForce RTX 4080. The system has 32GB of RAM and a 1TB mechanical hard drive. The programming language used is Python 3.9. The deep learning framework used is PyTorch 2.0.1. The GPU acceleration libraries used are CUDA 11.8 and CUDNN 8.8.0.

The learning rate of the network training is set to 0.0001, the batch size is set to 16, and the number of iterations is set to 200. Transfer learning can shorten the model training time. Therefore, we use the pre-trained weight file obtained from training the YOLOv8 model on the COCO2017 dataset as the initial weight file for training the wheat seed dataset. This helps accelerate network convergence and improve training performance.

## 3. Results

### 3.1. Performance of YOLOv8-HD

We conducted experiments on the wheat seed dataset using the YOLOv8 object detection algorithm, and the results are shown in Table 6. It can be observed that the detection performance is good in four scenarios: scattered without impurities, scattered with impurities, clustered without impurities, and clustered with impurities. However, YOLOv8 performs poorly in detecting stacked impurities, with a mAP of 68.5%. Therefore, this study focuses on proposing the YOLOv8-HD algorithm to improve the detection accuracy in scenarios with stacked impurities.

**Figure 10:**
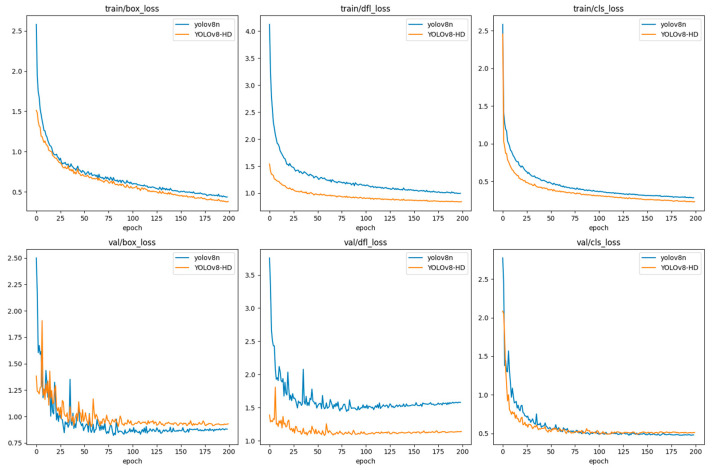
Training loss (**above**) and validation loss (**below**) curves of YOLOv8-HD and YOLOv8 models.

We compared the performance of the proposed YOLOv8-HD with YOLOv8 in cluttered scenes. The loss curves of the training and validation sets are compared in Figure 10.

We can see that both YOLOv8-HD and YOLOv8 have decreasing loss curves within 200 epochs until they stabilize. Additionally, YOLOv8-HD has a faster convergence on the training set, and the dfl_loss curve converges faster on the validation set compared to YOLOv8. This indicates that YOLOv8-HD is able to extract features more effectively, accelerating the convergence speed of the model.

**Figure 11:**
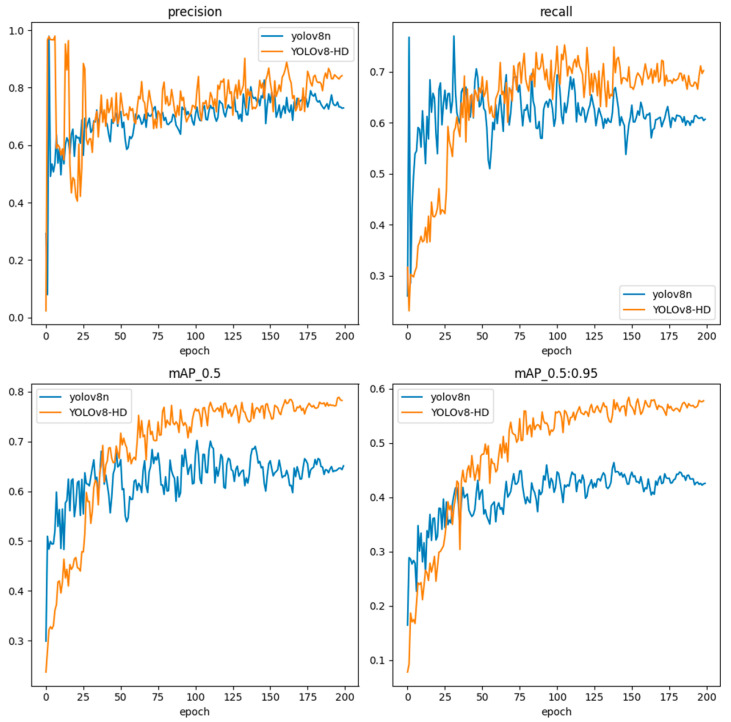
Performance curves of YOLOv8-HD and YOLOv8 models.

We compared the precision, recall, mAP_0.5_, and mAP_0.5:0.95_ of the proposed YOLOv8-HD and YOLOv8 in cluttered scenes over 200 runs and plotted the curves, as shown in Figure 11. From Figure 11, we can see that the proposed YOLOv8-HD outperforms the original YOLOv8 algorithm in terms of precision, recall, mAP_0.5_, and mAP_0.5:0.95_.

The specific detection results of wheat seeds using the YOLOv8-HD algorithm in stacked impurity scenes are shown in Table 7. From Table 7, it can be observed that the YOLOv8-HD algorithm proposed in this study achieves an average precision of mAP_0.5_ is 77.6% in stacked impurity scenes, which is a 9.1% improvement compared to YOLOv8. The mAP for wheat seeds, straw, and husk also improves, with improvements of 0.6%, 14.3%, and 12.3% respectively. The average precision of mAP_0.5:0.95_ is 58.2%, which is an 11.9% improvement compared to YOLOv8. The mAP_0.5:0.95_ for wheat seeds, straw, and husk also improves, with improvements of 2.8%, 19.8%, and 13.3% respectively.

The comparison of YOLOv8-HD and YOLOv8 detection results in cluttered scenes is shown in Table 8. Table 8 demonstrates that our proposed YOLOv8-HD not only improves the detection accuracy but also shows improvements in speed and model size. Specifically, the YOLOv8-HD model achieves precision, recall, mAP, and mAP_0.5:0.95_ of 84.5%, 70.8%, 77.6%, and 58.2% respectively, which are 7.4%, 4.5%, 9.1%, and 11.9% higher than YOLOv8. Additionally, the memory size of the YOLOv8-HD model is 6.35 MB, which is approximately 4/5 of YOLOv8. The GFLOPs of YOLOv8-HD decrease by 16%. The inference time of YOLOv8-HD is 2.86 ms (on GPU), which is lower than YOLOv8. These results indicate that our improvements on YOLOv8 significantly enhance the baseline accuracy and performance of wheat seed detection in cluttered scenes.

**Figure 12:**
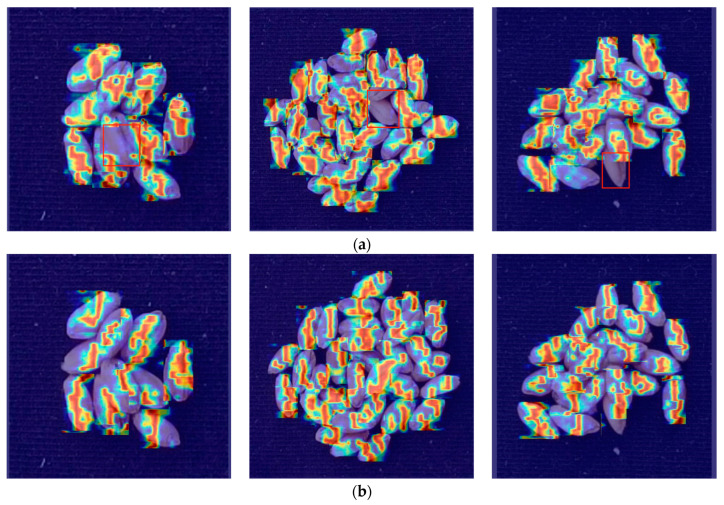
Comparison between YOLOv8-HD and YOLOv8 heatmaps: (**a**) YOLOv8 heatmaps, where the red boxes indicate regions with less prominent feature extraction.; (**b**) YOLOv8-HD heatmaps.

To better visualize the network’s feature extraction capability, we plotted the heatmaps of YOLOv8-HD and the original YOLOv8 algorithm for wheat grain detection, as shown in Figure 12. The red boxes in Figure 12a indicates a part where feature extraction is not prominent. From the image, it can be observed that the original YOLOv8 network exhibits weak feature extraction capabilities in cases of severe grain adhesion, making it prone to missed detections.

**Figure 13:**
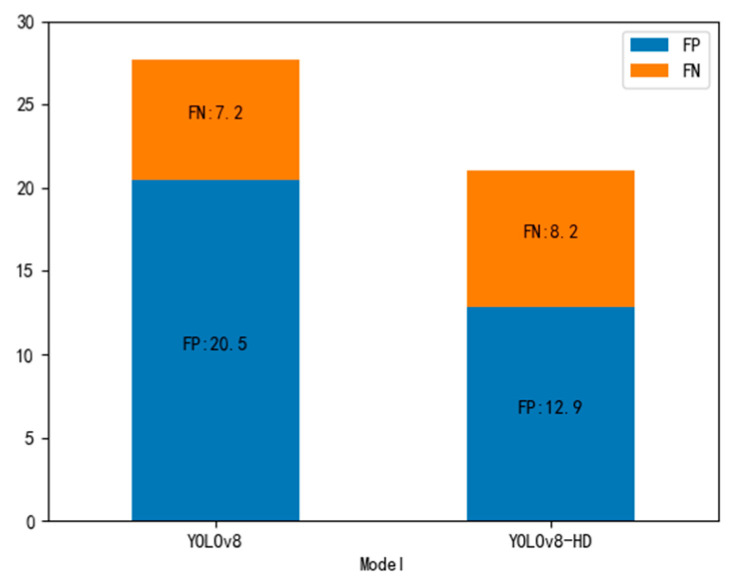
Comparison of FP/FN for YOLOv8-HD and YOLOv8.

We used TIDE to calculate the false positive and false negative rates of YOLOv8 and YOLOv8-HD models in wheat grain detection and plotted a bar graph for comparison, as shown in Figure 13. In Figure 13, FP represents False Positive, and FN represents False Negative. It can be seen that YOLOv8-HD significantly reduced the false positive rate but showed a slight increase in the false negative rate, indicating a major improvement in the false alarm rate with YOLOv8-HD but not a significant effect on the missed detection rate. However, in wheat grain statistics, false alarms have a greater impact, as they may result in impurities being counted as wheat grains.

To further validate the YOLOv8-HD algorithm proposed in this paper for wheat seed detection in practical operations, we conducted experiments by mixing wheat seeds in five different scenarios: scattered without impurities, scattered with impurities, clustered without impurities, clustered with impurities, and stacked with impurities. The experimental results are shown in Table 9. The YOLOv8-HD model achieved precision, recall, mAP, and mAP_0.5:0.95_ of 99.1%, 98.5%, 99.3%, and 89.2%, respectively, surpassing YOLOv8 by 17.2%, 14.8%, 16.8%, and 49%. This once again proves the effectiveness of the proposed YOLOv8-HD in improving wheat seed detection performance in various scenarios.

**Figure 14:**
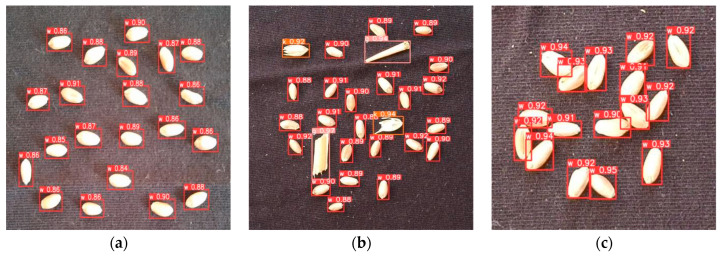

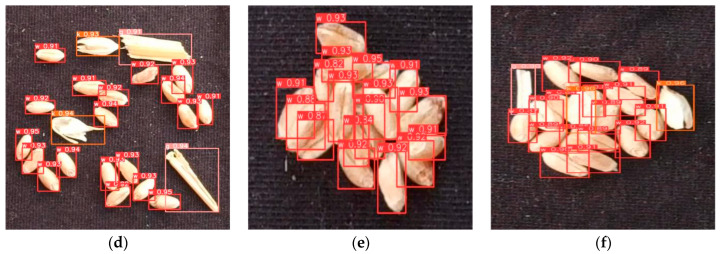
Visualization of YOLOv8-HD detection results: (**a**) dispersed clean; (**b**) dispersed cluttered; (**c**) clustered clean; (**d**) clustered cluttered; (**e**) clustered clean with cluttered; (**f**) clustered cluttered.

The visual detection results of the proposed YOLOv8-HD algorithm in dispersed clean, dispersed cluttered, clustered clean, clustered cluttered, and clustered clean with cluttered scenes are shown in Figure 14. It can be observed that the YOLOv8-HD algorithm can accurately detect wheat seeds in different scenes, and even in heavily imbalanced datasets of wheat husks and wheat straws, it can still effectively detect impurities such as husks and straws.

### 3.2. Ablation Experiments

We introduced a lightweight design for the detection head and the Vision Transformer with a Deformable Attention mechanism into the YOLOv8 network. To further validate the performance of the improved YOLOv8 model, this study sets up a series of ablation experiments to verify the performance of four different network configurations. The performance is shown in Table 10. In Table 10, D represents the Vision Transformer with Deformable Attention module, H represents the lightweight detection head module, YOLOv8-D represents the integration of the Vision Transformer with the Deformable Attention mechanism into YOLOv8 C2f, YOLOv8-H represents the network with a lightweight design detection head, and YOLOv8-HD represents the network formed by introducing both improvements into the YOLOv8 model. From a quantitative perspective, the performance of the four network configurations is analyzed, and objective evaluations are conducted on the test set of wheat seed images. Evaluation metrics include model detection accuracy and model parameters. It can be observed from Table 9 that the improved model achieves higher average precision compared to the other three models, and the model parameters are reduced compared to YOLOv8.

To further compare the performance of YOLOv8-D, YOLOv8-H, YOLOv8, and YOLOv8-HD networks, we used TIDE to compard the original YOLOv8 algorithm with the improved YOLOv8-D, YOLOv8-H, and YOLOv8-HD. The results are shown in Table 11. From Table 11, through lightweight design, YOLOv8-H shows a decrease in errors in Cls, Both, Dupe, Bkg, and Miss aspects but an increase in error in the Loc aspect. After adding the DAT module to YOLOv8-H, we observed a decrease in errors in Cls, Loc, Both, Bkg, and Miss. When the DAT module is added separately to YOLOv8, errors in Cls, Both, Dupe, and Bkg decrease. This analysis indicates that YOLOv8-H’s localization ability in wheat target detection is weakened, while YOLOv8-D’s classification ability in wheat target detection is enhanced, with a slight decrease in localization ability. By combining these two improvement methods, YOLOv8-HD shows enhanced classification and localization capabilities in wheat target detection.

### 3.3. Performance Comparison of Different Models

To quantitatively compare the performance of the improved model in the stacked wheat seed scene, the improved model was evaluated on wheat seed images in the test set, along with Faster R-CNN [43], YOLOv5, YOLOv7 [44], and the original YOLOv8 models. Table 12 presents the performance results of different detection models in the test set. From Table 12, it can be seen that compared to other models, the improved YOLOv8 wheat seed detection model performs the best in terms of mAP_0.5_ for wheat seeds, wheat stems, and wheat husks, with a value of 77.6%. This is an improvement of 11.8, 15.3, 13.1, and 9.1 percentage points compared to Faster R-CNN, YOLOv5, YOLOv7, and the original YOLOv8 models, respectively. The improved model in this study also achieved the best performance in terms of model memory consumption compared to the original YOLOv8 model, as it has undergone lightweight improvements.

To further quantitatively compare the detection performance of the improved model in all scenes for wheat seed detection, performance evaluations were conducted on wheat seed images using the improved model, Faster R-CNN, YOLOv5, YOLOv7, and the original YOLOv8 model. Table 13 presents the performance results of different detection models on the test set. From Table 13, it can be observed that compared to other models, the improved YOLOv8 wheat seed detection model achieves the highest mAP_0.5_ for wheat seeds, wheat stems, and wheat husks, which is 99.3%. This represents an improvement of 26.8, 18.7, 26, and 16.8 percentage points over Faster R-CNN, YOLOv5s, YOLOv7, and the original YOLOv8 model, respectively. The improved YOLOv8 wheat seed detection model also performs the best in terms of mAP_0.5:0.95_ and GFLOPs, providing strong evidence that the proposed YOLOv8-HD model has excellent performance for wheat seed detection.

### 3.4. Wheat Seed Counting

**Figure 15:**
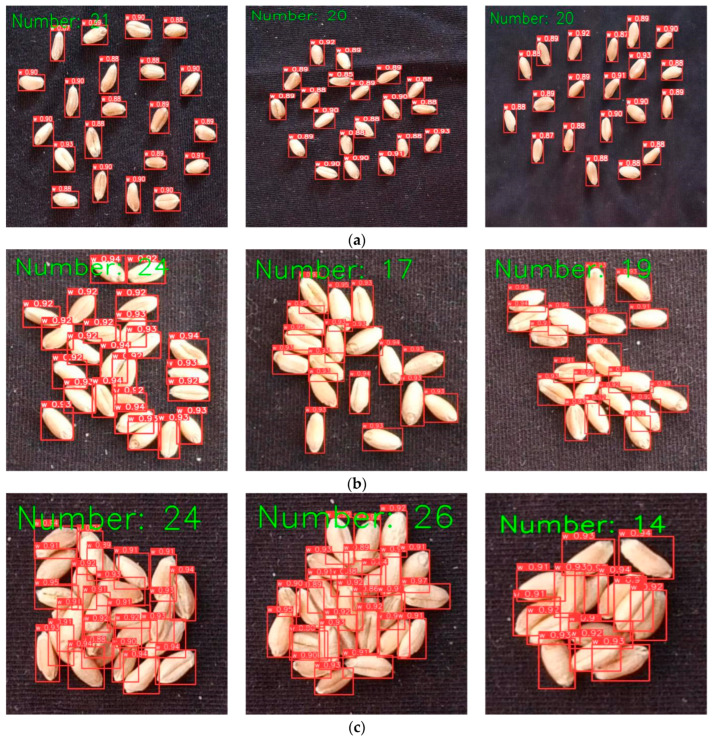
Wheat seed counting results: (**a**) wheat seed counting results in dispersed scenario; (**b**) wheat seed counting results in clustered scenario; (**c**) wheat seed counting results in stacked scenario.

Utilizing the YOLOv8-HD model, wheat seed counting in different scenarios was conducted, and the statistical results are shown in Figure 15. From Figure 15, it can be observed that the YOLOv8-HD model is capable of effectively detecting wheat seeds in dispersed, clustered, and stacked situations, accurately counting the number of wheat seeds within them.

## 4. Discussion

(1) When detecting wheat seeds, we compared the YOLO family algorithms, including YOLOv5, YOLOv7, and YOLOv8. We found that the YOLOv8 algorithm achieved the highest mAP (mean Average Precision) value of 82.5% in wheat seed detection. Additionally, it had the smallest model size and fastest running speed. Therefore, we selected YOLOv8 as the base algorithm for wheat seed detection.

(2) When using the YOLOv8 object detection algorithm to detect wheat seeds, we found that in stacked scenes, the wheat seeds are heavily occluded, resulting in poor detection performance. Therefore, we considered improving the YOLOv8 network structure to enhance the detection capability of wheat grains.

The Deformable Attention Transformer (DAT) is a general backbone network model with deformable attention. Its self-attention module can focus on relevant regions and capture more informative features, effectively improving the model’s sensitivity to small and dense targets, thus enhancing its detection capability. MS-Block, proposed in YOLO-MS [45], is a module for multi-scale feature fusion, which can effectively integrate features from different scales, enhance the detection capability for targets, and reduce the influence of background interference on recognition results. RFA [46] not only focuses on spatial features in the receptive field but also provides effective attention weights for large-size convolutional kernels, transferring attention from spatial features to receptive field spatial features. In this way, network performance can be further improved, leading to better results.

We added the above-mentioned DAT, MS-Block, and RFA to the C2f network of the YOLOv8 backbone network and compared their performance in wheat grain detection. The mAP values for wheat grain detection were 74.9%, 69.4%, and 52.1% for DAT, MS-Block, and RFA, respectively. It can be seen that DAT performs the best in wheat grain detection. Therefore, we adopted this method to improve YOLOv8 and enhance its detection capability for wheat grains.

Additionally, we incorporated the Swin Transformer, vanilla Transformer, and DAT attention mechanism into the base YOLOv8 model and tested them on the wheat grain dataset. The experimental results are shown in Table 14. From Table 14, it can be seen that the DAT attention mechanism achieves higher detection accuracy for impurities such as wheat straw and husks, indicating that the DAT attention mechanism has better handling capabilities for imbalanced datasets.

(3) In practical applications, wheat seed counters are designed to be small and portable, requiring the wheat seed detection model to have low memory usage and fast detection speed. Therefore, we considered a lightweight design for the YOLOv8 base model. We shared the convolutional parameters of the YOLOv8 detection head to reduce the parameter count and thus lighten the YOLOv8 detection head. As shown in Figure 5, we designed a shared 1 × 1 convolutional layer and a shared 3 × 3 convolutional layer for the YOLOv8 detection head. However, in our experiments, we considered multiple approaches: (a) sharing two 3 × 3 convolutional layers; (b) sharing two 3 × 3 grouped convolutions; (c) sharing one 1 × 1 convolutional layer and one 3 × 3 convolutional layer. The results of the three lightweight detection head methods are shown in Table 15. From Table 15, it can be inferred that considering a balance between detection accuracy and model size, scheme (c) achieves a relatively high mAP value while reducing the number of parameters in wheat seed detection. Therefore, scheme (c), which shares one 1 × 1 convolutional layer and one 3 × 3 convolutional layer, is adopted for the lightweight design of the YOLOv8 detection head.

We attempted other lightweight design approaches for YOLOv8 by replacing the YOLOv8 backbone network with Fasternet, named YOLOv8-Fasternet. Experiments were conducted on this dataset, and the results are shown in Table 16. From the experimental results, it can be observed that although YOLOv8-Fasternet reduces model size, it is inferior to the proposed YOLOv8-HD in terms of detection accuracy and GFLOPs, further demonstrating the effectiveness of our algorithm.

(4) To further validate the performance of our algorithm after lightweighting, we compared our algorithm with the lightweight model YOLOv7-tiny. The experimental results are shown in Table 17. From Table 17, it can be seen that YOLOv8-HD outperforms the lightweight object detection model YOLOv7-tiny in terms of detection accuracy, model size, and runtime speed.

Therefore, the YOLOv8-HD model demonstrates good performance in terms of accuracy, detection speed, and model size in wheat seed detection, making it easier to deploy on embedded platforms.

(5) To validate the model’s generalization ability, we conducted experiments on the global wheat ear dataset. The original YOLOv8 wheat ear detection mAP was 91.3%, with GFLOPs at 8.1. In this paper, the YOLOv8-HD wheat ear detection mAP was 95.7%, with GFLOPs at 6.8, indicating that the algorithm proposed in this paper has better detection capabilities.

## 5. Conclusions

We constructed a wheat seed dataset, including five different scenes: scattered without impurities, scattered with impurities, clustered without impurities, clustered with impurities, and stacked. By lightweighting the YOLOv8 detection head to improve the lightweight network architecture and incorporating the deformable attention transformer (DAT) into the YOLOv8 backbone network’s C2f layer to enhance the model’s detection accuracy, we named it the YOLOv8-HD network model. The YOLOv8-HD algorithm achieved an mAP of 77.6% in the stacked scene for wheat seed detection and an mAP of 99.3% across all five scenes, with a model inference time of 2.86ms. The YOLOv8-HD model has a smaller size and higher accuracy. Additionally, we compared the YOLOv8-HD model with mainstream object detection models, and the experimental results showed that the YOLOv8-HD model outperformed other networks in terms of mAP and model size. This ensures both detection accuracy and improved detection speed, which aligns with the deployment and application of agriculturally embedded devices, providing a wider range of application possibilities and technical support for the further development of wheat counting devices.

The proposed YOLOv8-HD model achieved wheat grain detection and counting in five different scenarios. However, in overlapping scenarios, the detection accuracy of wheat grains was only 77.6%, indicating room for further improvement. In the future, we will continue to optimize the model to enhance the performance of wheat grain detection in overlapping scenarios. Additionally, factors such as height and lighting were not considered in the data collection process, which may affect the model’s performance in real-world scenarios. We will further enrich the wheat grain dataset to facilitate its application in wheat grain counting.

## Figures and Tables

**Table 1:** 5 Scene divisions.

Scene	Description	Adhesion Condition
Dispersed without impurities	Each image contains scattered wheat seeds, with no adhesion condition	No adhesion
Dispersed with impurities	Each image contains scattered wheat seeds, with no adhesion condition, and there are impurities such as wheat straw and husk	No adhesion
Aggregated without impurities	Each image contains wheat seeds with mild adhesion phenomenon, and there are no impurities	Mild adhesion
Aggregated with impurities	Each image contains wheat seeds with mild adhesion phenomenon, and there are impurities	Mild adhesion
Stacked with impurities	Each image contains wheat seeds with severe adhesion phenomenon	Severe adhesion

**Table 2:** The number of annotations for different categories in the dataset.

Scene	Category	Total Annotations
Scattered without impurities	Wheat seeds	2065
Scattered with impurities	Wheat seeds	2261
	Straw	114
	Husk	360
Clustered without impurities	Wheat seeds	2296
Clustered with impurities	Wheat seeds	2860
	Straw	86
	Husk	147
Stacked with impurities	Wheat seeds	1949
	Straw	33
	Husk	72

**Table 3:** Dataset partitioning details.

Scene	Training Set	Validation Set	Test Set	Total
Scattered without impurities	420	120	60	600
Scattered with impurities	420	120	60	600
Clustered without impurities	420	120	60	600
Clustered with impurities	420	120	60	600
Stacked with impurities	420	120	60	600
Total	2100	600	300	

**Table 4:** The number of annotations for different categories after partitioning the dataset.

Scene	Category	Training Set	Validation Set	Test Set	Total
Scattered without impurities	Wheat seeds	8541	2402	1233	12,176
Scattered with impurities	Wheat seeds	9345	2620	1313	13,278
	Straw	477	142	61	680
	Husk	1062	290	161	1513
Clustered without impurities	Wheat seeds	9473	2642	1382	13,497
Clustered with impurities	Wheat seeds	11,834	3374	1641	16,849
	Straw	359	113	43	515
	Husk	603	187	81	871
Stacked with impurities	Wheat seeds	9275	1680	738	11,693
	Straw	120	36	42	198
	Husk	222	108	102	432

**Table 5:** The backbone network parameters of YOLOV8-HD.

Layer	Input	Operation	Parameters	Output
0	640 × 640 × 3	Conv	[16, 3, 2]	320 × 320 × 16
1	320 × 320 × 16	Conv	[32, 3, 2]	160 × 160 × 32
2	160 × 160 × 32	C2f	[32, True]	160 × 160 × 32
3	80 × 80 × 32	Conv	[64, 3, 2]	80 × 80 × 64
4	80 × 80 × 32	C2f	[64, True]	80 × 80 × 64
5	80 × 80 × 64	Conv	[128, 3, 2]	40 × 40 × 128
6	40 × 40 × 128	C2f	[128, True]	40 × 40 × 128
7	40 × 40 × 128	Conv	[256, 3, 2]	20 × 20 × 256
8	20 × 20 × 256	C2f_DAttention	[256, [20, 20], True]	20 × 20 × 256
9	20 × 20 × 256	SPPF	[256, 3, 2]	20 × 20 × 256

**Table 6:** Detection results of YOLOv8 in different scenarios.

Scene	Category	P/%	R/%	mAP_0.5_/%	mAP_0.5:0.95_/%
Scattered without impurities	Wheat seeds	99.9	0.988	99.5	83
Scattered with impurities	Wheat seeds	99.8	0.984	99.5	82.8
	Straw	1	0.971	99.4	82.5
	Husk	96.4	0.955	96.6	83.2
	Mean	98.7	0.97	98.5	82.8
Clustered without impurities	Wheat seeds	99.7	0.995	99.4	91.7
Clustered with impurities	Wheat seeds	99.7	0.988	99.5	90.3
	Straw	98.4	1	99.5	90.3
	Husk	98.9	98.6	99.4	88.7
	Mean	99	99.1	99.5	89.8
Stacked with impurities	Wheat seeds	92.7	98.8	98.5	74.8
	Straw	66.3	63.9	62.1	39.4
	Husk	72.3	36.2	45	24.6
	Mean	77.1	66.3	68.5	46.3

**Table 7:** YOLOv8-HD detection results.

Model	Scene		P/%	R/%	mAP_0.5_/%	mAP_0.5:0.95_/%
YOLOv8-HD	Stacked with impurities	Wheat seeds	91.7	99	99.1	77.6
		Straw	96	66.3	76.4	59.2
		Husk	65.9	47.2	57.3	37.9
		Mean	84.5	70.8	77.6	58.2

**Table 8:** Comparison of YOLOv8-HD vs. YOLOv8 in the stacked with impurities scenario.

Model	P/%	R/%	mAP_0.5_/%	mAP_0.5:0.95_/%	Model Size/MB	GFLOPs	Inference Time/ms
YOLOv8-HD	84.5	70.8	77.6	58.2	6.35	6.8	2.86
YOLOv8	77.1	66.3	68.5	46.3	7.67	8.1	3.47

**Table 9:** Comparison of YOLOv8-HD vs. YOLOv8 in all scenarios.

Model	Scene		P/%	R/%	mAP_0.5_/%	mAP_0.5:0.95_/%
YOLOv8-HD	5 types of scenes	Wheat seeds	99.8	99.1	99.4	89.7
		Straw	99.5	98.6	99.5	90
		Husk	98	97.9	98.9	87.8
		Mean	99.1	98.5	99.3	89.2
YOLOv8	5 types of scenes	Wheat seeds	98.9	91.9	97.8	45.7
		Straw	80.9	77.4	73.4	38.4
		Husk	65.8	81.9	76.5	36.6
		Mean	81.9	83.7	82.5	40.2

**Table 10:** Performance of the improved network.

Model	AP/%			mAP_0.5_/%	Parameters
	Wheat Seeds	Straw	Husk		
YOLOv8-D	98.8	63.9	62	74.9	3,902,297
YOLOv8-H	99	64.9	52.8	72.2	3,074,009
YOLOv8	98.5	62.1	45	68.5	3,834,521
YOLOv8-HD	99.1	76.4	57.3	77.6	3,214,169

**Table 11:** TIDE of the improved network.

Model	Cls	Loc	Both	Dupe	Bkg	Miss
YOLOv8	12.50	4.62	0.28	0.64	0.28	0.86
YOLOv8-D	9.22	4.72	0.11	0.05	0.25	2.12
YOLOv8-H	11.02	6.3	0.05	0.17	0.01	0.77
YOLOv8-HD	9.15	4.35	0.04	0.27	0.00	0.12

**Table 12:** Comparison of different algorithms in stacked cluttered scenes.

Model	AP/%			mAP_0.5_/%	Model Size/MB
	Wheat Seeds	Straw	Husk		
Faster r-cnn	98.5	48.6	50.3	65.8	108
Yolov5	98.9	56.8	31.2	62.3	14.4
Yolov7	98.9	45.1	49.6	64.5	74.8
YOLOv8	98.5	62.1	45	68.5	7.67
YOLOv8-HD	99.1	76.4	53.7	77.6	6.35

**Table 13:** Comparison of different algorithms in all scenarios.

Model	AP/%			mAP_0.5_/%	mAP_0.5:0.95_/%	GFLOPs
	Wheat Seeds	Straw	Husk			
Faster r-cnn	97.5	52.6	67.5	72.5	35.8	15.5
Yolov5	97.6	70	74.3	80.6	40.4	15.8
Yolov7	98.6	66.5	54.6	73.3	39.3	103.2
YOLOv8	97.8	73.4	76.5	82.5	40.2	8.1
YOLOv8-HD	99.4	99.5	98.9	99.3	89.2	6.8

**Table 14:** Comparison of different attention mechanisms.

Attention Mechanism	AP/%			mAP0.5/%
	Wheat Seeds	Straw	Husk	
vanilla Transformer	98.8	40	29	55.9
Swin Transformer	98.3	35.6	37.8	57.2
DAT	98.8	63.9	62	74.9

**Table 15:** Different schemes for lightweight detection head.

Schemes	AP/%			mAP_0.5_/%	GFLOPs	Parameters
	Wheat Seeds	Straw	Husk			
(a)	99	65.2	53.4	72.5	8.1	3,151,721
(b)	98.5	63.6	40.1	67.4	5.7	2,420,585
(c)	99	64.9	52.8	72.2	6.8	3,074,009

**Table 16:** Comparison of different lightweight methods.

Model	P/%	R/%	mAP_0.5_/%	mAP_0.5:0.95_/%	Model Size/MB	GFLOPs	Parameters
YOLOv8-HD	84.5	70.8	77.6	58.2	6.35	6.8	3,214,169
YOLOv8-Fasternet	54	63.1	55.9	31.3	6.21	10.7	4,175,869

**Table 17:** Comparison of different embedded models.

Model	P/%	R/%	mAP_0.5_/%	mAP_0.5:0.95_/%	Model Size/MB	GFLOPs
YOLOv8-HD	84.5	70.8	77.6	58.2	6.35	6.8
YOLOv7-tiny	61.5	55.6	53.6	28.9	12.3	13.2

## Data Availability

If scholars need more specific data, they can email the corresponding author.
